# Development and Evaluation of the Eetmaatje Measuring Cup for Rice and Pasta as an Intervention to Reduce Food Waste

**DOI:** 10.3389/fnut.2019.00197

**Published:** 2020-02-18

**Authors:** Corné van Dooren, Frederike Mensink, Kim Eversteijn, Marjolijn Schrijnen

**Affiliations:** Netherlands Nutrition Centre (Voedingscentrum), The Hague, Netherlands

**Keywords:** household food waste, measuring cup, portion size, intervention, cooking

## Abstract

Of all of the stages in the supply chain, more food waste comes from households than any other sector. A Dutch composition analysis showed that the solid food waste (including sauces, fats, and dairy products) from household waste amounted to 48.0 kg per person per year (in 2013), of which 5.0 kg consisted of cooked rice and pasta. These two product groups were numbers 1 and 3 in terms of relative waste: 34% of the purchased quantity of rice and 23% of that of pasta was wasted. Using questionnaires, we discovered that Dutch consumers mainly throw away food because they prepare too much of it. The same is true for rice and pasta because they increase greatly in volume when cooked. The water uptake ratio of rice is 2.5 (2.3–2.8) and that of pasta is 1.8 (1.5–2.3), which increases the chances of consumers overestimating portions. In 2013, more than half of the people surveyed did not measure pasta and rice portions. In view of this, the Netherlands Nutrition Centre developed a measuring cup called the Eetmaatje, which is marked with the recommended volumes for Dutch adults for different types of pasta and rice in terms of dry weight. The theoretical reduction of food waste the Eetmaatje provides is calculated to be ~6% for pasta and 21% for rice, or 12.5% combined. Between 2014 and 2019, more than 1.6 million Eetmaatje cups were distributed for free among Dutch households. Over that period, the measuring of pasta and rice by Dutch households increased. Most people (85–89%) in a panel of consumers who own an Eetmaatje think it is handy or very handy to use. The majority of those in the panel (50–80%) say that they use the Eetmaatje most times when they prepare a meal. Four out of five of those in the panel (77–87%) are convinced that the Eetmaatje helps them waste less pasta and rice. The Eetmaatje functions as a nudge to change cooking behavior and thus food waste behavior. Consumers who measure their pasta using the Eetmaatje self-reported that they produced less total food waste. The measured household waste of cooked rice and pasta seems to show a downward trend since the introduction of the cup. There is strong evidence that the Eetmaatje has increased the number of Dutch households measuring rice and pasta and thereby reducing food waste.

## Introduction

### The Problem of Waste

The Food and Agriculture Organization estimates that one-third of the world's food production is wasted (1). In the European Union, the annual per capita estimate is 173 kg of food waste per person, which is equivalent to Europeans wasting 20% of the continent's food production (2). Wageningen UR estimated the annual food waste in the Netherlands to be between 1,781 and 2,466 t, which is equivalent to between 105 and 145 kg per person (3). This figure covers all food lost and wasted from after primary production in the food industry to distribution to households. The group contributing the most to food waste is households (52%). According to these numbers, the focus in food waste reduction should be both on households and businesses, but with households prioritized. Consumer behavioral change should therefore be facilitated (4). In view of the above, the focus of this paper is on the reduction of avoidable household food waste. There are also large differences in amounts of food waste among countries (2). European households have the highest food waste numbers as measured in wasted kilocalories: 38% of total kCal (5). Food waste also contributes considerably to greenhouse gas emissions, land use, water use, fossil energy, and other inputs associated with food production (6). According to Tonini et al. (7), “Food preparation, for households and food service sectors, also provided an important contribution to the Global Warming impacts.” The United Nations placed food waste prevention on the international political agenda with the introduction of target 12.3 in the UN's Sustainable Development Goal 12 (Ensure sustainable consumption and production patterns): “By 2030, halve per capita global food waste at the retail and consumer levels and reduce food losses along production and supply chains, including post-harvest losses” (8).

### Food Waste by Consumers

Consumer-generated food waste is generated by multi-dimensional behavior, influenced by cultural, social, political, economic, and geographic factors, as well as cognitive, motivational, and structural factors, food-related behaviors, and food habits (9–11).

A compositional analysis of Dutch household food waste in 2016 showed that solid food waste (including sauces, fats, and dairy) from household waste amounts to 41.2 kg per person per year, which corresponds to 13% of the amount of food purchased. The annual waste of pasta and rice was measured to be 1.8 kg per person, or 4% of the total annual per capita waste. Rice and pasta are not the most wasted product groups in absolute amounts—they rank numbers 9 and 10, respectively, but in relative waste, they number 1 and 3 per product group: 34% of purchased rice and 23% of purchased pasta are wasted, both adjusted for water absorption during cooking in relation to the percentage of dry product purchased. A remarkable fact is that almost all of the wasted pasta and rice is cooked (12).

The Netherlands Nutrition Centre identified three main behavioral steps that can be taken to reduce consumer food waste: smart buying, smart cooking by using correct quantities, and better storage of food. These goals were defined and selected with the use of intervention mapping (13). Secondi et al. (14) concluded that a more precise measuring of portion sizes could potentially contribute substantially to reducing food waste. The main self-reported reason why Dutch consumers waste food is that they prepare more food than they consume (15, 16). Measuring portions could help reduce food waste in households.

### Possible Interventions

Stöckli et al. (17) recently reviewed waste-reduction interventions for consumers published in scientific and non-peer-reviewed reports. Their review concluded that “informational interventions are the most commonly used intervention type even though evidence indicates that this intervention type is relatively ineffective.” Interventions with a direct focus on food waste-related behaviors are supposed to be more effective. A four-country study shows that households reporting less waste tend to exhibit five food practices: planning of shopping and planning of meal preparation, exclusion of impulse buying, management of stocks and fridge, cooking the right quantities, and being creative with leftovers (18). A recent review of consumption-stage food waste reduction interventions found no effective interventions described on cooking the right portions or that little or no robust evidence was provided for the described interventions (19). Some effective interventions to promote buying smaller portion sizes in restaurant settings have also been identified, motivated by health reasons and food waste reduction (20). The low number of described interventions is worrying, according to Reynolds et al. (19), especially since most interventions suggested so far appear to be effective at reducing food waste. Nonetheless, most interventions do not focus on cooking and are not proven to be effective or reproducible.

**Figure 1:**
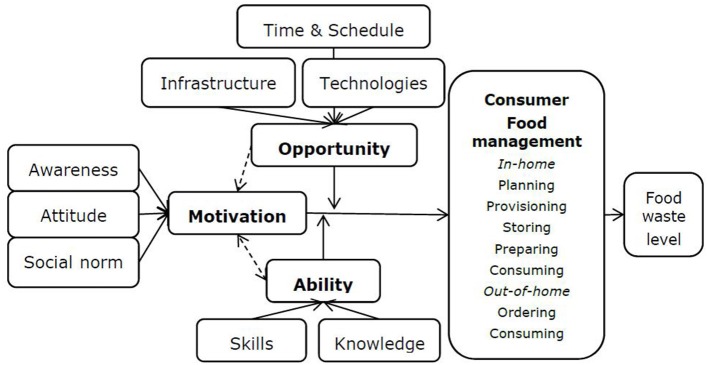
Consumer food waste model including the role of motivation, ability, and opportunity for predicting the level of food waste in households (18).

Information to consumers should be tailored to provide knowledge and skills to change particular food waste behaviors, ideally at the point of decision (21). Clear insights into factors related to consumer perceptions and behaviors related to food waste are necessary to reduce food waste in households (18). These factors are given in the consumer food waste model in Figure 1: consumer management of food waste related to preparing food is determined by motivation, opportunity, and ability, including skills and knowledge about portions (18). Aschemann-Witzel et al. (9) demonstrated that “consumers' motivation to avoid food waste, their management skills of food provisioning and food handling and their trade-offs between priorities have an extensive influence on their food waste behaviors.” Using interventions and experiments, it is possible to implement effective solutions, they concluded. This paper describes in detail the results of an intervention to promote cooking the right portions of pasta and rice.

### Aim

**Figure 2:**
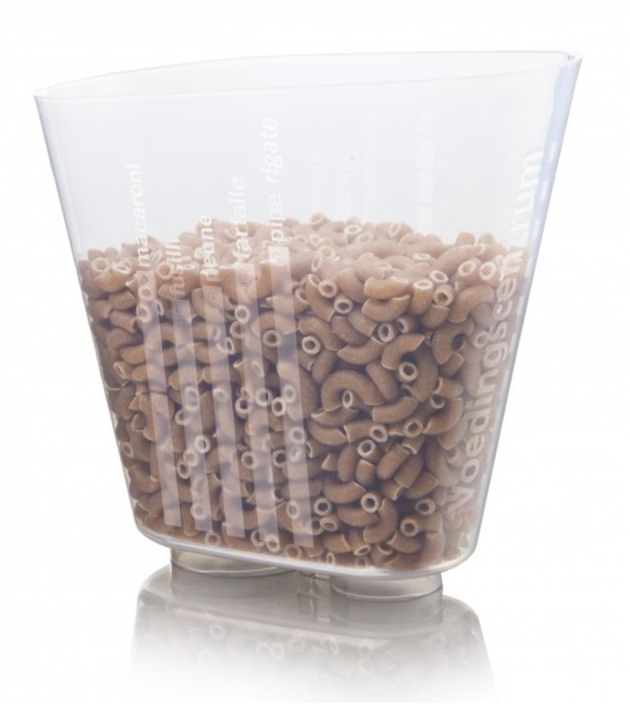
Measuring cup containing macaroni.

The aim of this paper is to perform an intervention on cooking the right amount of pasta and rice by using a measuring cup called the Eetmaatje (Figure 2) and to evaluate its contribution to cooking the right portions to reduce food waste in households. The cup reflects the recommended volumes for Dutch adults for different types of pasta and rice in terms of dry weight.

First, the paper provides a technical description of how to set the right uncooked portion sizes of pasta and rice for Dutch adults. Second, it argues how cooking the nutritionally recommended quantities can theoretically contribute to the reduction of food waste in most target groups. Third, we evaluate the use, satisfaction, and contribution to the reduction of food waste of the measuring cup by consumer research and by measuring actual food waste.

## Methods

This paper focuses exclusively on avoidable household food waste. We define food waste as food intended for human consumption that is not consumed (4, 22). Non-consumed food is split into avoidable and unavoidable food waste (5). Unavoidable food waste consists of the parts of food products that are not intended for consumption, such as shells, peels, stalks, cheese rinds, eggshells, coffee grounds, tea bags, meat bones, and fish bones (23).

Before and after the introduction of the Eetmaatje measuring cup, we conducted several consumer surveys. Most results and conclusions are based on the following four comparative, representative, biannual online questionnaires about food waste behavior in general:

° 2013—An ISO-certified survey with a 72% response rate, performed before the introduction of Eetmaatje by the GfK market research organization, involving a consumer panel of 2,055 adult main shoppers aged 18 or older (15).
° 2015—An ISO-certified survey with a 69% response rate, performed after the introduction of the Eetmaatje by GfK, involving a consumer panel of 2,054 adult main shoppers aged 18 or older (24).
° 2017—An ISO-certified survey with a 58% response rate, performed by GfK, involving a consumer panel of 2,010 main shoppers aged 18 or older (25).
° 2019—An ISO-certified survey with a 60% response rate, performed by Flycatcher Internet Research, involving a consumer panel of 997 main shoppers aged 18 or older out of 1,666 invited (26).

The questionnaires are designed to be comparable with one another (e.g., same question wording and order and same response options and wording). The four samples are stratified and representative of gender, age, region, and income, randomly sampled from a panel of 10,000 consumers. Differences between groups were tested with Chi-square tests, combined with z-tests for percentages and *t*-tests for averages. Significance was tested with 95% confidence. For averages, the maximum inaccuracy margin in this confidence interval is 3% (where *n* = 997 but lower than *n* > 2000). The Bonferroni method was used to correct multiple testing in order to reduce the possibility of significant differences by chance.

- Supportive evidence is based on a client panel of the Albert Heijn supermarket to evaluate the measuring cup in February 2014 (*n* = 336) and October 2014 (*n* = 330). These clients had the chance to receive an Eetmaatje for free in the supermarket in the winter of 2014. The client panel consisted of clients who were part of the supermarket's loyalty scheme and were randomly invited to take part in consumer research through a maximum of six questionnaires per year. Consumer research for Albert Heijn was carried out by Consumer & Business Insights Albert Heijn, Zaanstad. The two samples are independent.

Additional illustrative insights have been collected from:

- An online Facebook questionnaire among the visitors to the 2018 edition of Huishoudbeurs, a large annual fair for household products in Amsterdam, where 60,000 visitors received an Eetmaatje for free. The questionnaire resulted in *n* = 445 responses, mostly from women of low socio-economic status and therefore not representative of the total population.
- Professional clients of the Netherlands Nutrition Centre's web shop, such as dietitians and bodyweight coaches, among others, who ordered a package of 10 Eetmaatjes to distribute among their clients (*n* = 150).

Finally, we analyzed changes in household food waste between 2010 and 2019, measured by triennial sorting analyses in a representative sample of 130 Dutch households (23, 27, 28). The methodology and scientific protocol are described in detail in Van Dooren et al. (12).

## Results

### Development of the Measuring Cup

#### Setting the Portion Size

The recommended intakes of foods and food groups in the Netherlands were updated and published in 2016 (29). The recommended intakes of carbohydrate-rich products, including wholegrain products, such as pasta and rice, are summarized in Table 1. The recommendations are given in cooked serving spoons and in grams. A serving spoon of grain products is set as an average 50 g cooked, although other sources calculate 45 g for pasta and 60 g for rice (30). The portions are in line with earlier recommendations (Wheel of Five, 2004), except for the six spoons recommended for male adolescents. Although the recommendations are the same for adult men and women, we expect that the lower limit of 200 g fits women and people with a small appetite better, while the upper limit, 250 g, fits men and people with more appetite, in line with their metabolic energy needs. According to the Dutch National Food Consumption Survey (31), most pasta and rice is consumed during dinner. Nevertheless, part of the recommended intakes can be consumed on other eating occasions. Therefore, it was decided to apply cooked portions of ~200 g.

**Table 1:** Dutch recommended daily intakes of carbohydrate-rich products, including wholegrain products such as pasta and rice (29).

	**14–18 years old**	**19–50 years old (M + F)**	**51–70 years old (M + F)**
Recommendation in spoons (cooked)	4–5 spoons (F), 6 spoons (M)	4–5 spoons	3–4 spoons
Recommendation in grams (cooked)	200–250 grams, 300 grams	200–250 grams	150–200 grams

#### Water Absorption and Volume Ratios

The next step in our research was to translate cooked portions to quantities of dry product. The Netherlands Nutrition Centre recommends 100 g of dry pasta per person or 125 g for persons with a large appetite, which is equivalent to between 200 and 250 g of cooked pasta. The same double portion applies for rice: 75 g of dry rice or 100 g for people with large appetites translates into between 150 and 200 g of cooked rice. These figures are based on assumptions of water uptake of grain products during cooking. The official Dutch measurements and weights table calculates a factor of 2.5 for different types of rice and pasta (30), but the more recent food composition table of the Dutch Food Composition Database (NEVO) applies different factors between different kinds of cooked and uncooked grains: 2.65 for wholegrain pasta, 2.48 for white rice, and 2.72 for brown rice (32) (*personal communication Annette Stafleu, Netherlands Nutrition Centre, 16-7-2018*).

Some scientific research looking into the cooking properties of rice and pasta already exists. Thomas et al. (33) found that, for white rice, the water uptake ratio is 2.5 (with a range of 2.33–2.75). Steglich (34) studied the water absorption of spaghetti. The cooking process is characterized by steady water absorption: the longer the time, the more absorption. The best cooking time was 4 min with a 2.25 factor, while in a range of 3–5 min, the uptake ratio varied from 2.0 to 2.5 (34).

The biggest pasta producer in the world, Barilla, is one of the few that provides water absorption ratios for different kinds of pasta (35) (see Table 2). Portions of spaghetti are not measured in volume but in circumference, where 2 oz. of spaghetti corresponds to 5.4 cm in circumference (35). Based on these proportions, one 100 g portion of spaghetti for one person is 7.17 cm of spaghetti in circumference, while a two-person serving of 200 g will measure 10.14 cm.

**Table 2:** Water uptake factors between dry and cooked pasta.

	**Portion dry (in cups)**	**Portion cooked (in cups)**	**Water uptake factor**	**Dutch portion of 200 g cooked, calculated as dry (grams)**
Tortellini	0.5	1	2.0	100
Farfalle	0.75	1.25	1.7	120
Macaroni	0.5	1.125	2.3	89
Penne small	0.5	1	2.0	100
Penne average	0.66	1.25	1.9	106
Shells	0.75	1.125	1.5	133
Wholegrain penne/shells	0.75	1.125	1.5	133
Average			**1.83**	**112**

Table 2 demonstrates the water uptake factors for other pasta, ranging between 1.5 for shells and 2.3 for macaroni. The average is 1.83, which is somewhat lower than the 2.0 suggested by different sources. On average, preparing a one-person portion requires 112 g of dry pasta. The selected factor for pasta in the cup is 1.83.

Recipes available online and on the packages of the leading pasta and rice products on the Dutch market mostly use 100 g of dry carbohydrate product per person, as we recommend, but sometimes they use 75 g. Using 100 g dry pasta in recipes, which corresponds to a 2.0 factor, will result in a cooked quantity that is close to the average recommendation of 109 g dry pasta (factor 1.83).

#### Cup Design and Testing

Based on these factors (2.5 for rice and 1.83 for pasta) and recommendations (~200 g cooked), the Eetmaatje measuring cup was developed in cooperation with the Dutch Creative Brands Group, a company from Delfgauw, the Netherlands. This company specializes in the development, production, and distribution of innovative houseware products. The company contributed to the design of the cup and the selection of a food-safe, recyclable material (Figure 2). The portion sizes in volume for different types of pasta and rice were measured and tested by hand using cardboard molds, which were filled and weighed until the right volumes were calibrated. The company is the owner of the design and is responsible for its production and transport (see https://youtu.be/Hvp2B-jyOqg).

The difference in measured consumed volume between men and women (28, Table 3) was addressed by putting a simple message on the package: “This measuring cup indicates uncooked portions. These uncooked portions are based on the daily quantities of wholegrain cereals that the Nutrition Centre uses for an adult Dutch woman.” However, the 200 g portions were expected to be also adequate for most men or meet the actual average needs of male and female members of a household, as well as eaters with a small or large appetite.

**Table 3:** Theoretical reduction of rice and pasta waste by using the Eetmaatje measuring cup on consumption days.

	**Consumption (g)**	**Cooked (g)**	**Advised quantity (g)**	**Weekly reduction in waste**	
**Grains mixed**	**Median**	**(If 28% wasted)**	**Average rice/pasta**	**(grams)**	**(percentage)**
Men 19–30 y	172	239	185	54	29
Women 19–30 y	120	167	185	−18	−10
Men 31–50 y	172	239	185	54	29
Women 13–50 y	127	176	185	−9	−5
Men 51–70 y	168	233	185	48	26
Women 51–70 y	140	194	185	9	5
Average (unweighted)				**23**	**12**
**When only rice**		**(If 34% wasted)**	**Rice 75 g** **×** **2.5**		
Men 19–30 y	172	261	188	73	39
Women 19–30 y	120	182	188	−6	−3
Men 31–50 y	172	261	188	73	39
Women 13–50 y	127	192	188	4	2
Men 51–70 y	168	255	188	67	36
Women 51–70 y	140	212	188	24	13
Average (unweighted)				**39**	**21**
**When only pasta**		**(If 23% wasted)**	**Pasta 100 g** **×** 1.83^*^		
Men 19–30 y	172	223	183	40	22
Women 19–30 y	120	156	183	−27	−15
Men 31–50 y	172	223	183	40	22
Women 13–50 y	127	165	183	−18	−10
Men 51–70 y	168	218	183	35	19
Women 51–70 y	140	182	183	−1	−1
Average (unweighted)				**12**	**6**

* *Average water uptake (see Table 2)*.

### Theoretical Reduction of Rice and Pasta Waste

In this section, we approximately calculate the theoretical reduction of rice and pasta waste caused by using the measuring cup (Table 3). According to the Dutch National Food Consumption Survey 2007–2010 (31) the median consumption of pasta, rice, and other grain products excluding bread is 168–173 g for men and 120–140 g for women on consumption days (rice and pasta were consumed twice a week). The types of pasta and rice are not specified. Those quantities are lower than the recommended intakes. Table 3 summarizes the consumption of pasta and rice by population subgroups and consumption days. We added the average measured waste percentage to those quantities (27) in scenarios where they consumed only pasta (+23%), only rice (+34%), or half pasta, half rice (+28%). We then compared these amounts with the advised amount on the Eetmaatje measuring cup. The difference between the median cooked quantity and the amount advised on the Eetmaatje cup, which is the actual cooked amount when everyone uses the measuring cup, provides the result for the theoretical reduction of food waste. For the average adult, the approximate result is 6% for pasta and 21% for rice, which is equivalent to 12.5% combined. However, looking into the average for pasta and rice combined, it is theoretically possible that women between 19 and 50 years are still going to waste ~5–10% more. For all other groups, a reduction in food waste proportional to the result obtained is expected.

The portions used in the cup design were based on recommended and not on actual intakes, so the cup could lead to more food waste among consumers who actually eat less than the recommendations, but, in theory, we expect an overall reduction nonetheless. From a nutritional perspective, it is important for public health bodies to communicate the recommended quantities as part of food-based dietary guidelines (29) instead of promoting low quantities to accommodate people who simply eat less. The lower consumption from women is expected to be compensated for in households of two or three people by an expected higher intake from the other household members, who in most cases, are men. In this sense, the Eetmaatje measuring cup functions as an indicator of the needed quantity. We assume that the measuring cup does not influence the quantities people actually eat, only the amount they use to cook, but this cannot be entirely excluded.

### Measuring Portions Before the Introduction of the Cup

According to Temminghoff and Damen (15), almost half of the consumers say that they measure ingredients most times when preparing a meal, but only a fifth do this for every meal. Consumers in general do not know the right portion sizes per person, such as for rice. They randomly rely on intuition to measure pasta for cooking, for example, or they simply prepare an entire package of it at once. Households that do not use any kind of measuring during cooking report that they throw away more food than households that measure (15, 16), however other factors could explain these answers. Before the intervention started in 2014, close to half of Dutch consumers (41%) determined pasta quantities to cook based merely on their intuition or estimation by eye (15). This suggests that at least 41% of the population was likely to cook too much pasta and waste some of it, considering most pasta waste is generated from cooking. According to Temminghoff and Damen (15), a 12% share of consumers said they always cooked an entire package of pasta or rice, regardless of their actual needs or household size. This practice does not necessarily lead to waste if any excess rice or pasta cooked is eaten as leftovers at a later date. In families with children and youths aged between 6 and 17, the share of consumers saying they cook entire packages of rice or pasta rises to 16–17% (15). Other consumers already used an instrument to measure dry food quantity to cook before the introduction of the intervention, usually a kitchen scale (21%), a teacup (17%), or a measuring cup (6%) (15).

### Evaluation With Consumer Panels

After testing, design, and production, the Eetmaatje was introduced in February 2014. Within 2 weeks, one million items were distributed for free among customers of the biggest retailer in the Netherlands, Albert Heijn. The Dutch Minister of Food and Agriculture received the very first Eetmaatje, generating free publicity and consequently helping with distribution, along with advertisements. Shoppers received one Eetmaatje for free when they bought two packages of pasta or rice on sale, which was done to make sure that regular pasta or rice consumers were the ones receiving the free Eetmaatje cups. In the years that followed, another 0.6 million Eetmaatje cups were distributed through other channels, such as the web shop of the Netherlands Nutrition Centre, other supermarket chains, and the 2018 and 2019 edition of the Huishoudbeurs fair.

The market research company GfK and Flycatcher Internet Research carried out consumer research using independent, representative consumer panels, before introduction (2013) and biannually thereafter, in 2015, 2017, and 2019. The results are summarized in Table 4. Before the introduction of the Eetmaatje in 2013, only 6% of the people surveyed said they used some sort of measuring cup to prepare pasta. By 2015, 2 years after the introduction of the cup, this share doubled to 12%. In 2017, more than half of this share (7%) were using the Eetmaatje, as opposed to none in 2013 (25). In 2019, the share of consumers using the Eetmaatje cup was 8%. Table 4 shows that some consumers (4%) shifted between 2013 and 2019 from a teacup to another type of measuring. Those who used a traditional measuring cup or a teacup did not switch to the Eetmaatje, while using a scale to weigh food quantities actually increased in 2017. The group of people not measuring dry food before cooking decreased from 53% in 2013 to 46% in 2019, showing significant decreases in both groups using random by-eye estimation and those cooking entire packages at once (2017). The research shows that one-person households and families without children under 18 tend to use a measuring cup, a teacup, or a scale to measure dry food to cook more often. Women more frequently weigh dry food amounts (28%), while men use a teacup more often (18%). Among wealthy consumers, the percentage of those who weigh the amount of dry food they cook is higher (32%) (25).

**Table 4:** Consumers who measure and do not measure pasta, including whether they measure dry food using a scale, a teacup, a measuring cup, or the Eetmaatje (before and after the introduction of the Eetmaatje), 2013–2019.

	**GfK (15)**	**GfK (24)**	**GfK (25)**	**Flycatcher (26)**	
	**2013 (*n* = 2055) (%)**	**2015 (*n* = 2054) (%)**	**2017 (*n* = 2010) (%)**	**2019 (*n* = 997) (%)**	**Trend**
Quantity estimated by eye	41	37	39	35	Sign. decrease 2019
Whole package cooked	12	13	9	11	Sign. decrease 2017
Total no measuring	**53**	**49**	**48**	**46**	
Weighing with scale (grams)	21	20	23	26	Sign. increase 2017
Measuring by teacup	17	16	15	13	
Measuring by measuring cup	6	12	12	10	Sign. increase 2015
Of which Eetmaatje (no 2015 data)	0	?	7	*8*	Sign. increase 2017
Total measuring	**44**	**47**	**49**	**49**	
Others/unknown	3	4	3	5	
Percentages adjusted for total of 100%	**100**	**100**	**100**	**100**	

*?, not measured*.

We, together with Albert Heijn, conducted two evaluation surveys among the retailer's client panel, the first in February 2014, just after the Eetmaatje's introduction (*n* = 336), and the second in October 2014, 8 months after the introduction (*n* = 330). According to the results of these surveys, the Eetmaatje is most commonly used for rice (72%), followed by pasta (50%). The second questionnaire showed that 30% of Albert Heijn's customers know of the cup, among which 59% own one, which is equivalent to 17% of the entire panel. Familiarity with the cup in the panel decreased significantly to 30% from 42% 8 months after the first questionnaire, but ownership remained comparable at 17 vs. 20%, which is not a significant drop.

**Figure 3:**
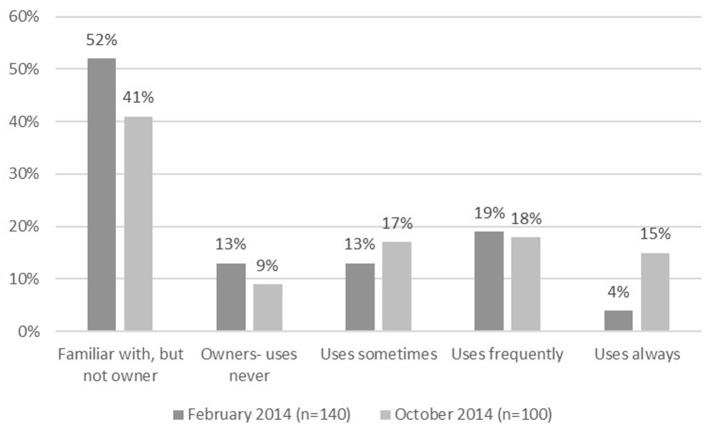
Eetmaatje ownership and frequency of use, February and October 2014 (36).

Among owners, a 26% (15 of 59% owners) share said they always use the cup, 30% (18/59%) use it frequently, and 28% use it sometimes (17/59%). The “always” category increased from 8% (4/48%) in the first questionnaire to 26% (15/59%) in the second, while the “never” category decreased from 27% (13/48%) to 16% (9/59%) (Figure 3). Within the total samples, the numbers of frequent users were, respectively, *n* = 67 and *n* = 59 in each survey. Their reported reasons for using the Eetmaatje were always cooking the right portions, generating less food waste, using it being easier than weighing dry food, and eating healthy portions. For the majority of users, the portions of the Eetmaatje are right (66%), but for 15%, they are too small, and for 3%, too big. The most important barriers cited by non-users were that the amounts do not match the desired quantity, a preference for weighing or using a different measuring cup, or forgetting to use the Eetmaatje. A majority of owners in the panel (88%) reported that they were very positive about the Eetmaatje cup, 87% said they are convinced that it helps them cook the right portions, and 77% said they are convinced that it helps them reduce food waste. These results are comparable to the results from the first questionnaire: 85% positive, 89% convinced about the right portions, 83% convinced about food waste reduction [(36), not published]. Albert Heijn is, with a 35% market share, the biggest supermarket chain in the Netherlands. Although their client profile may differ from other chains, the self-reported food waste from Albert Heijn clients does not differ significantly from clients from other chains (37).

A recent questionnaire among visitors to the Huishoudbeurs fair in 2018, which is not representative of the entire Dutch population, confirmed these results. The recent questionnaire shows that 59% always use the Eetmaatje, 28% frequently use it, 5% sometimes use it, and 13% never use it. A share of 87% of the respondents was convinced that the tool helps them reduce their food waste in terms of pasta and rice.

The Eetmaatje was also distributed through the web shop of the Netherlands Nutrition Centre. Dietitians and bodyweight coaches, among other professionals, could order packages of 10 Eetmaatjes to give to their clients for free. In 2017, 150 of those professionals responded to a questionnaire, which provided additional insights: 90% of them recommend the Eetmaatje to overweight clients. Another 53% recommend it to clients with healthy weights and 30% to underweight clients. The Eetmaatje is mainly advised to determine correct portion sizes (89%), but other reasons were also frequently cited, such as losing weight (49%), reducing food waste (46%), and ensuring that the right amount is consumed (43%). For 90% of the professionals surveyed, the Eetmaatje delivers the desired results, meaning accurate portion sizes and eating according to food-based dietary guidelines.

### Measuring Actual Household Food Waste

**Figure 4:**
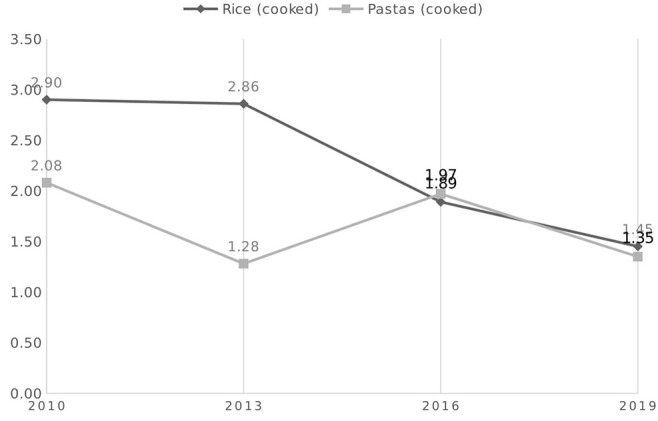
Measured annual waste of cooked rice and pasta in collected household food waste in the Netherlands before 2010 and after the introduction of the measuring cup in 2013, 2016, and 2019, in kg per capita.

CREM Waste Management measured actual food waste in Dutch households in 2010, 2013, 2016, and 2019 (12, 16, 23, 27, 28). These measurements are longitudinal: every time 130 households from the same 13 districts and streets were sampled. Total food waste showed a significant downward trend from 48 kg to 47.4 kg to 41.2 kg to 34.3 kg. Rice and pasta ranked numbers 9 and 10 on the list of most wasted products in 2016. Figure 4 shows a downward trend in wasted amounts of rice and pasta before and after the introduction of the Eetmaatje. Before the introduction (2010), the average annual per capita waste of cooked rice and pasta from households was 2.9 kg for rice and 2.1 kg for pasta. Although not statistically confirmed, there seems to have been a decrease in the waste of rice, halving to 1.45 kg, but the decrease of pasta is less clear, fluctuating through the years and ending in 2019 at 1.35 kg. This downward trend cannot be directly attributed to the introduction of the Eetmaatje, since part of the wasted rice is from take-out meals, and the reduction could be the result of other interventions.

Food waste was also measured in 2016 by a self-reporting frequency questionnaire (38). Self-reporting gives an underestimation relative to waste measured by sorting analysis (39). Respondents were asked about their agreement with the statement: “Within our household, we try as much as possible to weigh/measure ingredients.” Answers were related to their reported food waste. Respondents who weigh their ingredients as well as possible wasted less food than respondents who do not. The difference in self-reported food waste between the highest and lowest groups was 16.8 kg less food waste per year. This correlation—of households that weigh food wasting less—does not necessarily mean it is causal, because it could also be explained by an overall awareness about reducing food waste in those households.

## Discussion

### Ability to Change Behavior

Awareness of the environmental and moral consequences of food waste does not directly correlate with the amount of reported food waste in households (18). This study demonstrates that additional factors stimulate consumers to use a measuring cup:

- Usability and convenience of the measuring cup compared to weighing
- Choosing the right, healthy portions
- Losing weight

In line with the model of Van Geffen et al. (18), convenience and health support consumer motivation. The Eetmaatje cup contributes to the ability of the consumer to cook precise, healthy portions. The health and convenience aspects of using the Eetmaatje meet the consumer's sustainability goals and the common-sense social norm not to waste food.

Most human behaviors are habits, which are less susceptible to rational change. This implies that policies aimed at changing habitual behaviors need to consider the strength of habits and the difficulty of establishing new ones and breaking existing ones. Establishing new habits might include helping people who intentionally want to break the habit, such as through information prompts (40). The Eetmaatje could help to create a new habit, viewed as a prompt to remind the consumer to cook precise portions and reduce waste. From the literature, we know that a higher frequency of cooking is likely to improve skills in, for example, cooking the right portions (41, 42). Using the Eetmaatje measuring cup leads to better matching of individual appetites and circumstances in the household. In practice, food waste reduction is expected, especially in the half of Dutch consumers who have not used measuring instruments or scales so far, proportionally contributing more to rice and pasta waste.

The general feeling of having the ability to change behavior has been examined under the related terms self-efficacy and perceived behavioral control (43). Stancu et al. (44) found perceived behavioral control to have a significant effect on self-reported food waste behavior. In addition to this general feeling of control, the present study has examined the feeling of portion control, i.e., specific abilities to cook precise portions and how they contribute to reducing food waste, in more detail. It is necessary for consumers to be convinced that they can change their own behavior. From earlier research, we can conclude that perceived behavioral control reduces food waste in households (44). The present study underlines that it is essential for interventions to support self-efficacy.

In their review of interventions, Stöckli et al. (17) found that prompts in general were relatively more effective at changing behavior compared to informational interventions. Osbaldiston and Schott (45) defined prompts as “verbal or written messages designed to remind people to perform a target behavior.” Nudges are a relatively new phenomenon in the field of reducing food waste (17). Nudges such as changes to plate type and size as well as portion size already demonstrated that they can lead to a reduction in food waste out of the home (46, 47). But there is no evidence that prompts and nudges to reduce food waste are effective in households. Our paper adds evidence to this field. The Eetmaatje could be viewed as a kind of prompt in the kitchen, helping people to cook more precise portions, as well as a nudge to remind the consumer about reducing food waste every time they cook.

### Portions in Other Countries

It is interesting to look at the possibilities of implementing the Eetmaatje outside the Netherlands. Other countries most likely have other recommendations for pasta and rice, depending on the culture, food, and energy needs of the population. In Italy, a common portion of cooked pasta is ~105 g (35). The British Nutrition Foundation recommends cooked medium portion sizes of 180 g for rice, 230 g for pasta or macaroni, and 220 g for spaghetti (48). The US consumption is in the range of 120 to 175 g (49). The Union of Organizations of Manufacturers of Pasta Products of the European Union refers to cooked portion sizes between 180 and 220 g (50). In conclusion, the Eetmaatje could be used in other countries but may require small adjustments in portion sizes.

### Possible Improvements

Users (see http://liefdevoorlekkers.nl/2014/02/06/eetmaatje/) suggested that the Eetmaatje is also applicable for other types of grain products that are less frequently used. For example, bulgur and pearl barley seem to have the same water absorption properties as rice and quinoa, while polenta (cornmeal) and buckwheat have the same properties as couscous. Oat and other breakfast cereals would also be possible suggested uses for the Eetmaatje. These are all good possibilities for further improvement of the Eetmaatje, but their inclusion should be further supported by literature or tests. Another user-suggested improvement could be switching the choice of material to compostable or bio-based plastic.

The literature indicates that the water uptake factor of brown rice is higher (2.7–3.9) than that of white rice (2.5) (33). Although the consumption of white rice is currently much higher than that of brown rice (31), food-based dietary guidelines advise an increase in the consumption of brown rice. In the future, a separate measure for brown rice could be added to the Eetmaatje cup.

The Eetmaatje cup is designed for adult portions, but they are also applicable for adolescents between 14 and 18 years old. The development of a version for children could be investigated, as could adding instructions on how to apply adult portions sizes to children, for example, 1 adult portion = 2 children portions (up to a certain age).

Although several studies found statistical correlations between factors and food waste, it is important to understand the theory that explains these correlations (21). Policy-makers who are responsible for consumer-focused interventions and the experts assisting them should therefore strive to identify evidence for causal relationships before they develop, implement, and evaluate interventions for reducing consumer food waste (17, 51). After our study, the REFRESH project published guidance for evaluating interventions preventing household food waste (52). The Eetmaatje intervention is categorized as “prompting people to undertake desired behavior” with a theory-based and an empirical impact evaluation, including measuring outputs, intermediate outcomes, and final outcome. Looking at the recommendations, our intervention could be improved, for instance, by establishing an evaluation plan before the intervention in order to have a better control group and reference measurement.

An estimation of the eventual waste reduction achieved with the use of the Eetmaatje could be done. The theoretical annual waste reduction is ~6% or 624 g per person for pasta (Table 3: 12 g/week) and 21% or 2028 g per person for rice (39 g/week). Based on a distribution of 1.6 million Eetmaatje cups, which are frequently used by at least 50% of receivers who have an average household size of 2.1 persons, the annual waste reduction could be at least 1,050 t of cooked pasta or 580 t uncooked and 3,410 t of rice or 1,360 t uncooked. These are approximate calculations, suggesting that waste reduction could in fact be lower or higher. Figure 4 shows a downward trend in cooked pasta waste of 0.73 kg per person (12,600 t) and in rice waste of 1.45 kg per person between 2010 and 2019 (25,100 t for the population). The changes appear too large to be attributed to the Eetmaatje alone. The 8% who reported the use of the Eetmaatje in 2019 (Table 4) corresponds with half of the maximum of 20% of the 7.9 million households that could own a cup. Many different factors may have affected consumer food waste behavior; in the last decade awareness campaigns on environmental sustainability and other interventions have been performed that may have also influenced this behavior.

## Conclusions

Less than half of Dutch consumers measured the portions of dry pasta and rice for cooking before the Eetmaatje measuring cup was introduced. Measuring portions and use of the Eetmaatje increased in the 6 years after introduction. There is strong evidence that the Eetmaatje has increased the number of Dutch households measuring rice and pasta and thereby reducing food waste. Using recommended portions is expected to reduce waste from cooking pasta and rice. The Eetmaatje cups distributed in our panels remain in use by 50–80% of the consumers who received one, while 85–89% Eetmaatje owners are satisfied with the tool, considering it useful. Approximately 80% of users report that the Eetmaatje cup helps them cook precise, healthy portions and waste less pasta and rice. In addition, consumers who measure pasta before cooking produce less total food waste, according to self-reports. The Eetmaatje measuring cup functions as a nudge toward changing cooking behavior, consequently helping to reduce food waste. In 2019, the measured actual household waste from cooking pasta and rice in the Netherlands showed a downward trend compared to 2013, before the intervention, which does not show that the reduction is directly related to the intervention. In the future, the Eetmaatje tool could be applied to other products and in other countries. Reducing food waste is not the only motivation for consumers to adopt the Eetmaatje measuring cup, but other factors, such as convenience and cooking healthy portions, should also be promoted.

## Data Availability Statement

The datasets generated for this study are available on request to the corresponding author.

## Author Contributions

CD wrote the paper. FM and KE were responsible for the consumer research and questionnaires. MS managed the project. FM, KE, and MS contributed to the content of the paper and gave critical inputs.

### Conflict of Interest

The authors declare that the research was conducted in the absence of any commercial or financial relationships that could be construed as a potential conflict of interest.
